# Elucidating the Molecular Determinants of the Binding Modes of a Third-Generation HIV-1 Integrase Strand Transfer Inhibitor: The Importance of Side Chain and Solvent Reorganization

**DOI:** 10.3390/v16010076

**Published:** 2024-01-02

**Authors:** Qinfang Sun, Avik Biswas, Dmitry Lyumkis, Ronald Levy, Nanjie Deng

**Affiliations:** 1 Center for Biophysics and Computational Biology and Department of Chemistry, Temple University, Philadelphia, PA 19122, USA; qinfang.sun@temple.edu (Q.S.); ronlevy@temple.edu (R.L.); 2 Laboratory of Genetics, The Salk Institute for Biological Studies, La Jolla, CA 92037, USA; abiswas@salk.edu (A.B.); dlyumkis@salk.edu (D.L.); 3 Department of Physics, University of California San Diego, La Jolla, CA 92093, USA; 4 Graduate Schools for Biological Sciences, Section of Molecular Biology, University of California San Diego, La Jolla, CA 92093, USA; 5 Department of Chemistry and Physical Sciences, Pace University, New York, NY 10038, USA

**Keywords:** INSTIs, naphthyridine, binding modes, molecular dynamics, free energy method, conformational free energy, side chain reorganization, solvent reorganization, conformational entropy

## Abstract

The first- and second-generation clinically used HIV-1 integrase (IN) strand transfer inhibitors (INSTIs) are key components of antiretroviral therapy (ART), which work by blocking the integration step in the HIV-1 replication cycle that is catalyzed by a nucleoprotein assembly called an intasome. However, resistance to even the latest clinically used INSTIs is beginning to emerge. Developmental third-generation INSTIs, based on naphthyridine scaffolds, are promising candidates to combat drug-resistant viral variants. Among these novel INSTIs, compound **4f** exhibits two distinct conformations when binding with intasomes from HIV-1 and the closely related prototype foamy virus (PFV) despite the high structural similarity of their INSTI binding pockets. The molecular mechanism and the key active site residues responsible for these differing binding modes in closely related intasomes remain elusive. To unravel the molecular determinants governing the two distinct binding modes, we applied a novel molecular dynamics-based free energy method that utilizes alchemical pathways to overcome the sampling challenges associated with transitioning between the two bound conformations of ligand **4f** within the crowded environments of the INSTI binding pockets in these intasomes. The calculated conformational free energies successfully recapitulate the experimentally observed binding mode preferences in the two viral intasomes. Analysis of the simulated structures suggests that the observed binding mode preferences are caused by amino acid residue differences in both the front and the central catalytic sub-pocket of the INSTI binding site in HIV-1 and PFV. Additional free energy calculations on mutants of HIV-1 and PFV revealed that while both sub-pockets contribute to binding mode selection, the central sub-pocket plays a more important role. These results highlight the importance of both side chain and solvent reorganization, as well as the conformational entropy in determining the ligand binding mode, and will help inform the development of more effective INSTIs for combatting drug-resistant viral variants.

## 1. Introduction

HIV-1 strand transfer inhibitors (INSTIs) are important components of antiretroviral therapy (ART) [1,2]. INSTIs work by blocking the catalytic function of the enzyme IN, which assembles into an oligomeric complex with viral DNA known as the intasome and is responsible for inserting viral DNA into the host cell’s DNA during the early stages of HIV-1 replication. This process is referred to as integration. By inhibiting catalytic integration, INSTIs effectively prevent the establishment of a provirus and further replication within the cell. However, HIV-1 resistance to even the latest clinically used INSTIs is beginning to emerge, as evidenced by accumulating mutations in the IN gene [3]. In recent years, a novel group of naphthyridine-based third-generation INSTIs has emerged, which demonstrate improved potency against drug-resistant variants of HIV-1 [4,5].

**Figure 1:**
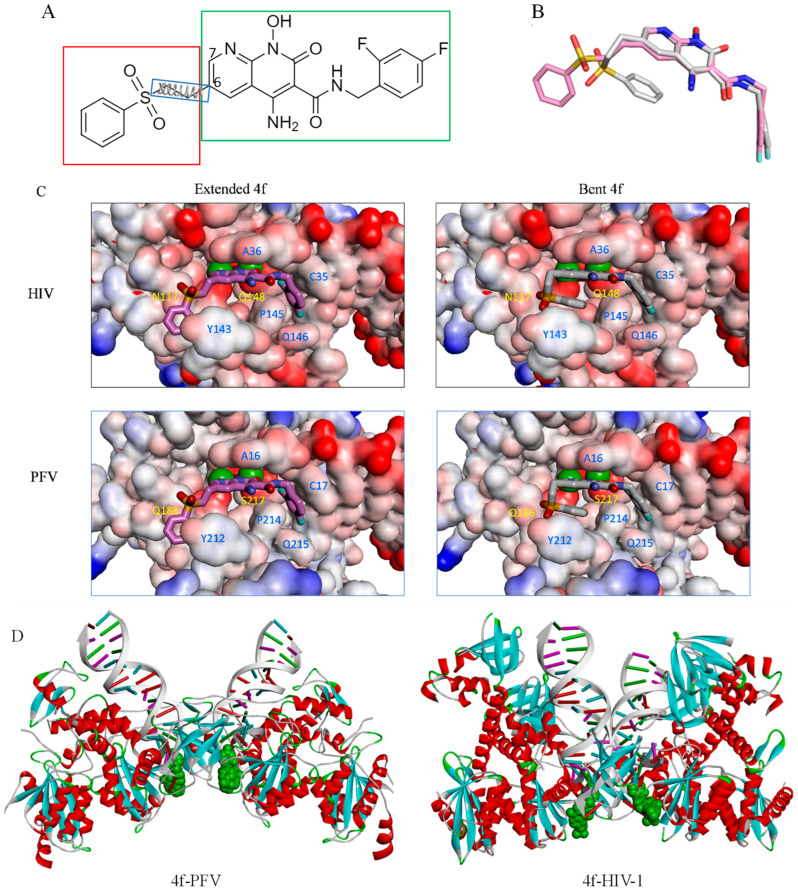
(**A**) Chemical structure of **4f**, consisting of a sulfonylphenyl group (red box), a naphthyridine core, and a 1,3 difluorobenzene group (green box). The three harmonic dihedral angle restraints that govern the conformation of the sulfonylphenyl group in the R-FEP-R simulations are also indicated(blue box). (**B**) Overlay of the two binding modes of **4f**: extended (pink) and bent (gray) binding modes. (**C**) **4f** bound to HIV-1 (upper) and PFV (lower) in the extended (left) and bent (right) conformations. The magnesium ions are shown as green spheres in these figures. (**D**) Overall structures of **4f** in complexes with the PFV (left) and HIV-1 (right) intasomes. The ligand **4f** is shown as green spheres.

The development of clinically used and developmental INSTIs has, in part, relied upon structural insights gained from intasome assemblies, which are targeted directly by this group of drugs. The structure of the prototype foamy virus (PFV) intasome with bound INSTIs was determined in 2010 [6], and this complex has been accordingly used extensively for structure-based drug design efforts [4,7,8,9]. However, recent structures of HIV-1 intasomes with bound INSTIs have revealed that some INSTIs can bind distinctly to intasomes from HIV-1 in comparison to intasomes from the related PFV. Specifically, one of the naphthyridine-containing compounds, **4f** (Figure 1A), which has exhibited superior potency in inhibiting drug-resistant viral variants in comparison to clinically used INSTIs [4,10], stands out by displaying distinct binding modes for these two intasomes (Figure 1B) [4,11]. Despite the high degree of similarity in both shape and amino acid composition within the INSTI binding pockets of the two intasomes, the 6-substituted sulfonylphenyl moiety, a critical component that contributes to the potency of naphthyridine compound **4f** [4,10], engages in intramolecular stacking with its own naphthyridine core within the PFV binding site of the PFV intasome, leading to a “bent” conformation of the ligand. In contrast, when this same ligand is bound within the pocket of the HIV-1 intasome, the sulfonylphenyl moiety adopts an extended conformation (Figure 1C). The differences in binding mode lead to distinct interpretations when analyzing mechanisms of drug resistance or when using the structural insights for the purposes of ligand modification, depending on whether the PFV or the HIV-1 model is being used to guide the modeling and ligand optimization.

We were interested in understanding the underlying reasons for the distinct binding modes observed experimentally. Little is known about the physical factors that stabilize the same ligand molecule differently inside binding pockets that are very similar; the RMSD between C-alpha atoms of corresponding binding site residues in the HIV-1 and PFV intasomes is ~0.5 Å (Table S1, Supporting Information). While variations in the amino acid residues that surround the bound INSTI and DNA substrates are believed to affect the binding of the ligand in the active site, it is unclear which of these residues are most responsible for altering the binding modes. Furthermore, while the structure of the HIV-1 intasome with **4f** bound was derived using cryo-electron microscopy (cryo-EM), the structure of the PFV intasome with **4f** bound was derived using X-ray crystallography. Whether the observed ligand binding mode is influenced by the conditions under which the experiment was performed (e.g., crystal packing or differences in buffers employed) remains to be examined and helped to motivate this modeling project.

A molecular understanding of the determinants for the INSTI’s binding modes in the two similar viral intasomes could provide insights to help improve inhibitor design. However, accurately estimating the relative thermodynamic stability of different binding modes for a flexible ligand inside the highly packed semi-enclosed space of the binding pocket is known to be computationally challenging [12,13,14,15]. While enhanced sampling methods such as metadynamics can be used to connect the two end-state binding conformations via physical pathways [13], identifying the relevant reaction coordinate to be used in such methods can be nontrivial, as it will involve correlated motions of both ligand and protein side chains to facilitate the conformational transition between the two binding modes inside the binding pocket. The resulting high dimensionality of the conformational free energy surface that needs to be explored can sometimes preclude convergence of the free energy estimation. In contrast to enhanced sampling methods that use pre-defined physical reaction coordinates to connect two conformational basins, the R-FEP-R method (restrain–free energy perturbation–release) [16] uses a dual topology alchemical pathway to circumvent the high free energy barriers that may arise from both intramolecular and intermolecular interactions in a crowded environment. R-FEP-R has been successfully applied to compute the free energy of conformational changes in protein loops [16] and DNA base pairing [17]. Here, we apply this method to estimate the conformational free energy difference between the two binding modes of the INSTI **4f** in complexes with either HIV-1 or PFV intasomes.

The results of the free energy calculations from the R-FEP-R method confirmed that the two distinct conformational binding modes of **4f**, one to PFV and the other to HIV, observed using the different experimental techniques, namely cryo-EM and X-ray crystallography, are indeed thermodynamically favored in solution and are not the result of the experimental conditions employed. Analysis of the MD structures revealed that changes induced by **4f** binding, in regard to the side chain conformations and the hydration patterns within the central sub-pocket, as well as changes in the torsional entropy in the front sub-pocket of the INSTI binding site, largely explain the relative thermodynamic stabilities of the different binding modes. Based on these structural insights, we performed further R-FEP-R free energy calculations on the different mutants of the two intasomes. The results show that these mutations will indeed significantly change the relative stabilities of the two binding modes in directions consistent with our physical explanation. Our study identifies the important residues responsible for the distinct way that **4f** binds to PFV compared with HIV and explains how ligand-induced side chain and solvent reorganization, as well as conformational entropy, affect the mechanisms of binding mode selectivity in the two closely related intasomes.

## 2. Results

### 2.1. The Calculated Conformational Free Energies Recapitulate the Preference for the Experimental Binding Modes

Previous high-resolution structural biology studies have indicated that compound **4f** can adopt either a bent or extended conformation when bound to the active sites of retroviral intasomes [4,11]. As shown in Figure 1C, the differences in the binding modes of **4f** when bound to either the HIV-1 or PFV intasome are largely confined to the orientation of the sulfonylphenyl group, which is appended to the 6-position of the naphthyridine core, while the remaining parts of the ligand largely superimpose in the two binding modes. The sulfonylphenyl group is connected to the naphthyridine core by three flexible dihedral angles: see Figure 1A. When the compound **4f** is bound to the HIV-1 intasome, the sulfonylphenyl moiety adopts an extended conformation, but when this same compound is bound to the PFV intasome, the sulfonylphenyl moiety adopts a bent conformation and forms an intramolecular stacking interaction with the planar naphthyridine core (Figure 1C). The shapes of the **4f** binding pockets in the two intasomes are very similar (Table S1, Supporting Information).

In principle, the conformational preferences of the ligand in the two intasome active site pockets may be captured by running long MD simulations initiated from either of the binding modes, and if the simulation time is sufficiently long, both binding modes will be sampled according to the Boltzmann factor of their relative free energy. However, this approach of running brute force MD simulations is ineffective because of the high free energy barriers from both intra-molecular and inter-molecular interactions. For example, no binding mode conversion is observed in multiple 30 ns MD simulations starting from either of the binding modes. A much better strategy in these situations is to use enhanced sampling methods like adaptive umbrella sampling [18] and metadynamics [19] to overcome the free energy barriers in conformational space. In these approaches, the key is to choose a suitable set of collective variables that defines a transition pathway connecting the two end states; in the present system, it will be particularly difficult for such calculations to converge, because the physical conformational pathways would involve large movements of not only ligand dihedral angles but protein side chain and backbone torsions. As seen in Figure S1, Supporting Information, to facilitate the conformational transition between the two binding conformations, both the Pro142 backbone and the Y143 side chain must rearrange to avoid steric clash with the sulfonylphenyl moiety of **4f** in order to convert from the extended to bent conformation, and vice versa.

**Figure 2:**
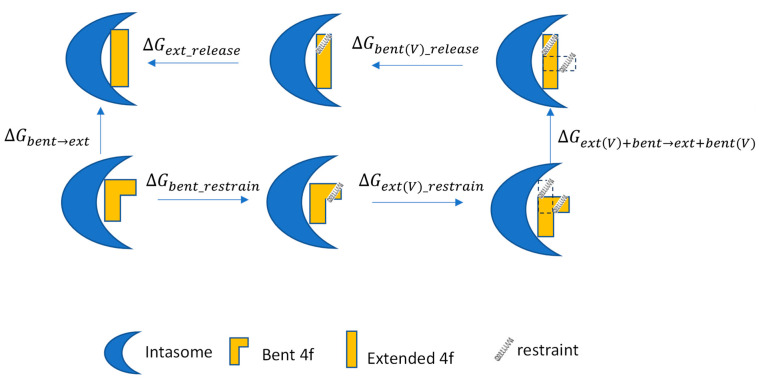
The R-FEP-R thermodynamic cycle for computing the conformational free energy difference of two binding modes. The letter V in the parenthesis indicates that the set of atoms in the dual topology set is virtual. The conformational restraints are represented by the springs.

Therefore, to avoid such possible complications, we chose to apply the recently developed R-FEP-R thermodynamic cycle, where the two end states, i.e., the extended and bent conformations of **4f**, are connected via an alchemical pathway (Figure 2) [16]. In this approach, the intermediate system consists of both the extended and bent sulfonylphenyl groups of the ligand **4f** using a dual topology, with their relative contributions to the Hamiltonian varied by the continuously varying alchemical λ parameter. In order to observe the alchemical disappearance/reappearance of the two sulfonylphenyl groups, the convergence of the free energy calculation must be facilitated by a symmetrical restrain and release cycle, in which the three dihedral angles in the flexible linker between the sulfonylphenyl group and the **4f** core (Figure 1A) are harmonically restrained to the values corresponding to each of the two end states.

Table 1 shows the R-FEP-R calculated conformational free energy difference $∆G_{bent\to ext\;}^{site}$ . For the wild-type (WT) HIV-1 intasome, the extended binding mode is favored with a −2.9 kcal/mol conformational free energy difference relative to the bent conformer. In contrast, when bound to the PFV intasome, the extended **4f** conformer is disfavored by 2.2 kcal/mol relative to the bent **4f**. Thus, these calculations correctly account for the experimentally observed binding modes, which provides validation for the physical models employed here. The result also supports the inference that the differing binding modes of **4f** to intasomes from HIV-1 or PFV are due to differences in the two nucleoprotein complexes, specifically, rather than to the experimental conditions under which the structures were determined, i.e., X-ray crystallography vs. cryo-EM.

### 2.2. Understanding the Mechanism for the Binding Mode Selectivity in the Two Intasomes: Analysis of MD Structures and Further R-FEP-R Free Energy Calculations

To gain insights into the molecular determinants that impact the selection of the different binding poses by the two intasomes, we analyzed in detail the 30 ns MD trajectories of the INSTI binding site in the different **4f** binding modes.

**Figure 3:**
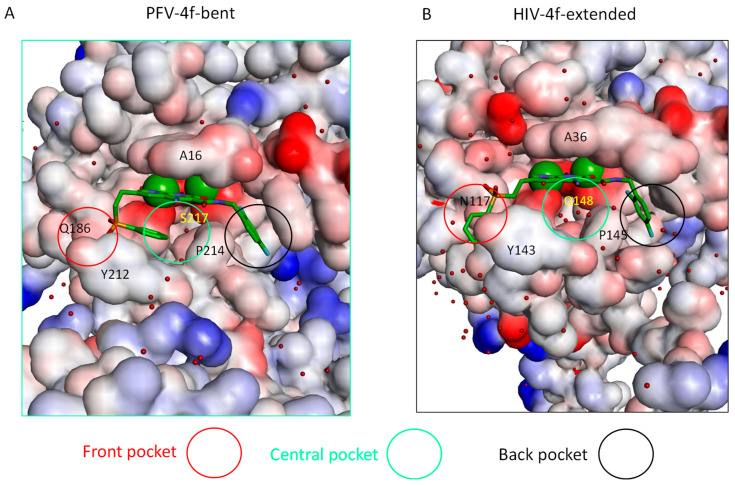
The three sub-pockets (front, central, and back) in the complexes of (**A**) bent **4f**-PFV (left) and (**B**) extended **4f**-HIV-1 (right) INSTI binding sites. The water molecules are shown as red spheres. The magnesium ions are shown as green spheres. Note that in the extended binding mode, the sulfonylphenyl group occupies the front pocket, leaving the central pocket solvated, whereas in the bent binding mode, the front pocket is solvated and the central pocket is partially occupied.

**Figure 4:**
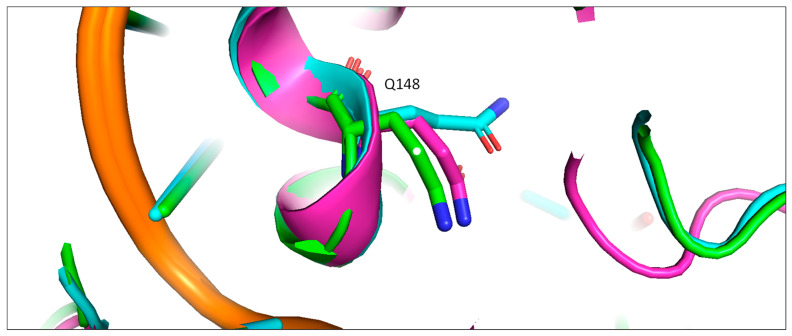
The rotamer states of Q148 of HIV-1 in the apo and **4f** bound states. Pink: apo; green: extended **4f** bound; blue: bent **4f** bound.

**Figure 5:**
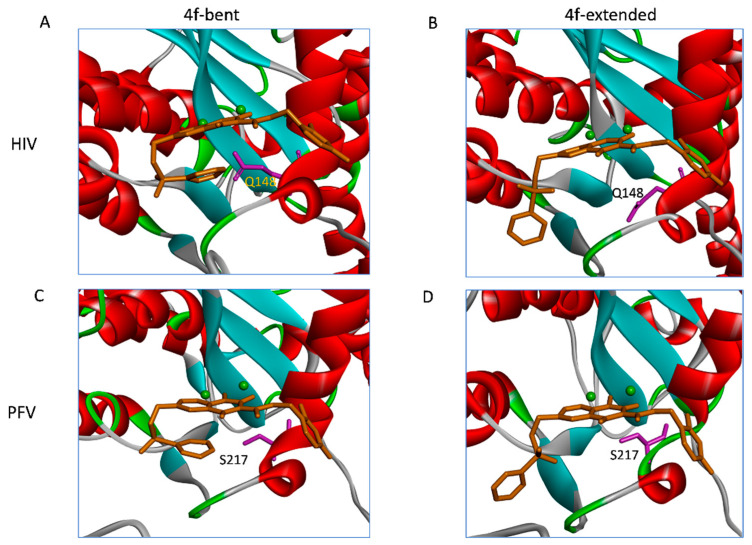
Upper: conformations of the Gln148 in the extended (**A**) and bent (**B**) binding modes in the HIV-1 intasome. Lower: conformations of the Ser217 in the extended (**C**) and bent (**D**) binding modes in the PFV intasome. The two magnesium ions are shown as green spheres.

In HIV-1 and PFV intasomes, the two distinct conformations of **4f** are characterized by the manner in which the sulfonylphenyl group of **4f** interacts with two sub-pockets, which we refer to as the front pocket (HIV-1: Asn117/Tyr143/Pro142; PFV: Gln186/Tyr212/Pro211) and the central pocket (HIV-1: Pro145/Gln148; PFV: Pro214/Ser217): see Figure 3. In the extended binding mode, the sulfonylphenyl group of **4f** occupies the front pocket, while the central pocket is filled with solvent. In contrast, in the bent binding mode, the central pocket is partially occupied by the sulfonylphenyl group, leaving the front pocket exposed to the solvent (Figure 3). Comparing the binding site residues (within 5 Å from any **4f** atoms; see Table S1, Supporting Information) at equivalent positions in the two intasomes, we find the following differences: HIV-N117 vs. PFV-Q186 in the front pocket and HIV-Q148 vs. PFV-S217 in the central pocket. Analysis of the MD trajectories reveals distinct conformations and hydration patterns involving HIV-Q148 vs. PFV-S217 in the central pocket (see also Table 2 and Table 3 and Figure 4 and Figure 5). Below, we will first discuss the consequences of the HIV-Q148 vs. PFV-S217 difference in the central pocket with regard to the binding mode selection, followed by discussing the role of HIV-N117 vs. PFV-Q186 in the front pocket.

### 2.3. The Central Sub-Pocket Is Primarily Responsible for the Binding Mode Selection by the Two Intasomes

We first consider how the compound **4f** engages the HIV-1 intasome in its two distinct binding modes. In the extended binding mode, the sulfonylphenyl group of **4f** occupies the front pocket and forms favorable intermolecular π−π stacking interactions with Tyr143 of HIV-1 (Figure 3). In this binding mode, the central pocket is occupied by solvent, allowing the side chain of Gln148 to adopt a conformation close to the apo-state conformation (Table 2 and Figure 4) and is well solvated by ~6 water molecules (Table 3 and Figure 3). In the bent binding mode, the sulfonylphenyl group folds back and partially occupies the central pocket, making a favorable intramolecular π−π stacking interaction with the naphthyridine ring system of **4f**; however, the favorable intermolecular π−π stacking with the front pocket residue Tyr143 is lost. Importantly, the central pocket is now partially occupied by the sulfonylphenyl moiety of **4f**, leading to its nearly complete desolvation. As the number of water molecules hydrating the central pocket drops from ~5–6 to 0, the resulting loss of favorable interactions between water and the polar side chain of Gln148 substantially destabilizes the bent binding mode. Furthermore, the partial occupation of the central pocket by the sulfonylphenyl group of the bent **4f** causes the Gln148 side chain to rearrange and adopt a high free energy conformation that is distinct from that of the apo-state (see Table 2, Figure 4 and Figure 5A). As we have previously shown [20], such ligand-induced conformational reorganization in the receptor is generally associated with a free energy penalty and results in decreased thermodynamic stability of the corresponding ligand-bound state [12,20,21,22].

We next consider how compound **4f** engages the PFV intasome in its two distinct binding modes. As in the **4f**-HIV-1 complexes discussed above, the extended binding mode is stabilized by the intermolecular π−π stacking between the sulfonylphenyl group and the front pocket residue, in this case Tyr212 of PFV, whereas the bent binding mode is primarily stabilized by the favorable intramolecular π−π stacking interaction between the sulfonylphenyl group and the naphthyridine ring system of **4f**. However, unlike the **4f**-HIV-1 complexes, here, the difference in the protein environment within the central pocket in PFV shifts the thermodynamic balance in favor of the bent binding mode. In the PFV intasome, the residue corresponding to HIV-1 Gln148 is Ser217 (Figure 1C). Serine has a shorter and less polar side chain in comparison to glutamine. As a result, when **4f** engages the PFV intasome in the bent conformation, the central pocket residue Ser217 can maintain the same conformation that is observed when **4f** engages the PFV intasome in an extended conformation (Figure 5B). In addition, because of the smaller size of the sidechain of Ser217, the central pocket remains weakly solvated by 1–2 water molecules even when the sulfonylphenyl group of the bent **4f** partially occupies this space (Table 3 and Figure 3). Thus, the bent **4f** in the PFV intasome active site is expected to lead to a smaller conformational reorganization free energy, and to a smaller desolvation free energy cost when compared with that in the HIV-1 intasome, where the corresponding central pocket residue is Gln148.

Overall, these analyses suggest that there are two primary determinants governing the binding mode selection by the HIV-1 and PFV intasomes: (i) there is a desolvation free energy cost to the bent conformation of **4f** in the HIV-1 intasome when the central pocket residue is Gln148; in contrast, there is a smaller free energy penalty for desolvation in the case of PFV, where the corresponding residue is Ser217; (ii) there is also an associated side-chain reorganization of Gln148 in HIV-1, which is not observed in the corresponding residue Ser217 in PFV. Collectively, these observations explain why the extended binding mode is favored in the **4f**-HIV-1 intasome complex, whereas the bent binding mode is more stable in the **4f**-PFV intasome complex, as captured via structural biology.

To test our hypothesis that the central pocket determines the binding mode preference, we generated in silico substitutions and performed R-FEP-R calculations on the Q148S mutant of the HIV-1 intasome and the S217Q mutant of PFV (Table 1). If Q148 in HIV-1 is indeed causing the extended binding mode to be more stable, then mutating this residue to serine, which is the corresponding residue in PFV IN, is expected to significantly reduce the relative thermodynamic stability of the extended binding mode over the bent mode. Similarly, if S217 in PFV is primarily responsible for the binding mode selectivity to favor the bent mode, then mutating this residue to a Glutamine should make the extended binding mode substantially more stable. Table 1 shows the results of R-FEP-R calculations on these mutants. In the HIV Q148S mutant, the calculated free energy difference $∆G_{bent\to ext\;}^{site}$ still favors the extended binding mode, but the calculated free energy difference is just − $0.7\pm 0.3\frac{kcal}{mol}$ , which is significantly smaller than the $∆G_{bent\to ext\;}^{site}=-2.9\pm 0.4kcal/mol$ observed with the WT HIV-1 intasomes. In fact, since the value of $∆G_{bent\to ext\;}^{site}\sim \;0.7\;kcal/mol$ for HIV Q148S translates to a population ratio of extended and bent **4f** of ~3:1, we accordingly designate these as “mixed” populations. Similarly, in the R-FEP-R simulation of the PFV S217Q mutant, switching the serine to glutamine caused a more dramatic change in free energy, such that the binding mode preference was actually reversed to slightly favor the extended binding mode over the bent binding mode. In both cases, the single point mutation causes significant changes in the binding mode stability by ≥2.2 kcal/mol, in the expected directions. These free energy results therefore support the conjecture that the Gln148 (HIV-1)/Ser217 (PFV) pair in the central pocket is a key factor in determining the binding mode preference in the two intasomes.

### 2.4. The Front Sub-Pocket Makes a Comparatively Smaller Contribution to the Binding Mode Selection by the Two Intasomes

**Figure 6:**
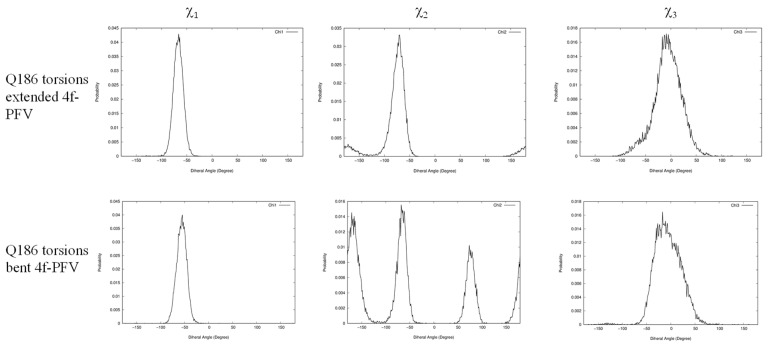
Distributions of side chain dihedral angles of Q186 of PFV in the two **4f** binding modes. All data are extracted from 30 ns MD simulations of the corresponding complexes.

**Figure 7:**
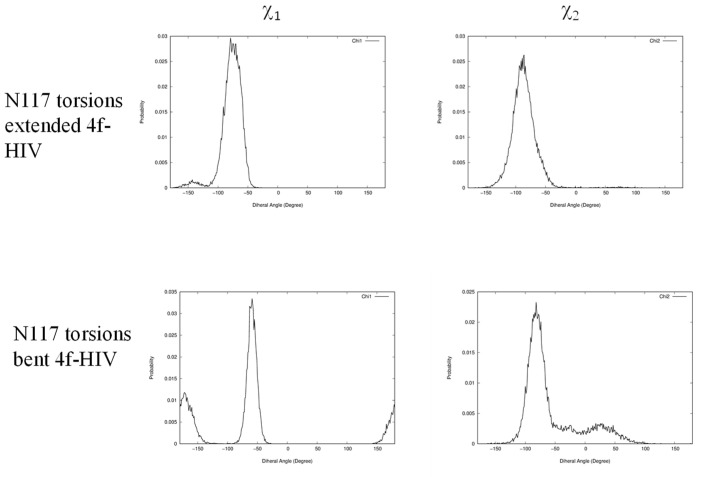
Distributions of side chain dihedral angles of N117 of HIV in the two **4f** binding modes. All data are extracted from 30 ns MD simulations of the corresponding complexes.

In addition to identifying the important role of central pocket residues Gln148 (HIV-1) and Ser217 (PFV), we also examined how the front pocket residues Asn117 (HIV-1) and Gln186 (PFV) impact the binding mode preference. In the extended **4f** binding mode, the nonpolar Cβ atoms of Asn117 of HIV-1 and Gln186 of PFV form similar hydrophobic interactions with the sulfonylphenyl ring (see Figure S2, Supporting Information), whereas in the bent binding mode, such interactions are absent because the front pocket is unoccupied. Importantly, the length of the side chain matters and Gln186 (PFV) is longer than Asn117 (HIV-1) by one rotatable bond. Consequently, when the ligand **4f** binds in the extended binding mode, placing the sulfonylbenzene ring into the front pocket, the side chain of Gln186 of PFV experiences a larger ligand-induced conformational entropy loss than the side chain of Asn117 of HIV-1 does. This can be observed by the side chain torsion angle distributions observed in the MD trajectories (Figure 6 and Figure 7). Specifically, in the extended binding mode, the conformation of the side chain dihedral angle χ_2_ of Gln186, PFV, is restricted and resides within a single basin (Figure 6, upper panel), but in the bent binding mode, it samples all three conformational basins (Figure 6, lower panel). This translates to a free energy contribution of $∆G_{bent\to ext}(Q186-PFV)\approx -kT\ln3=0.65\;kcal/mol$ , i.e., destabilizing the extended binding mode in PFV relative to the bent binding mode [23]. In HIV-1, however, the corresponding residue is Asn117, which has a shorter side chain. Asn117 largely occupies a single dominant basin in both the extended and bent binding modes of **4f** (Figure 7), giving rise to a smaller entropy loss that does not significantly impact the relative thermodynamic stability of the two **4f** binding modes.

To further probe the relevance of the residues within the front pocket, we performed R-FEP-R free energy calculations on both the HIV N117Q mutant and the Q186N mutant of PFV (Table 1); both mutations are in the front pocket. In the HIV N117Q mutant, the conformational free energy difference $∆G_{bent\to ext\;}^{site}=-2.5\pm 0.4kcal/mol$ , compared to $∆G_{bent\to ext\;}^{site}=-2.9\pm 0.4\;kcal/mol$ for wild-type HIV. Thus, mutating Asn117 to glutamine indeed destabilizes the extended binding mode of **4f** by ~0.4 kcal/mol, as predicted by our analysis based on the torsional entropy analysis. Similarly, in the PFV Q186N mutant, the bent binding mode is destabilized by ~0.8 kcal/mol by mutating Gln186 to Asn, again consistent with our entropy analysis of Gln186 vs. N117. These results therefore suggest that, in addition to the prominent role of the central pocket residues Gln148 (HIV-1) and Ser217 (PFV), the distinct residues Asn117 (HIV-1) or Gln186 (PFV) in the front pocket also contribute and help to explain the binding mode selection by the two intasomes, even though this effect is smaller than the effects induced by the mutations (HIV-1 Q148S and PFV S217Q) in the central pocket.

## 3. Discussion

### 3.1. Implications of the Findings for Drug Resistance and Ligand Binding

The findings here have relevance to our understanding of the role of mutations in and around the active site of IN for drug resistance and ligand binding. For example, our simulations suggest that the mutation N117Q in HIV-1 IN, which would be expected to yield viable viruses without significantly compromising enzyme activity [24,25], maybe a drug-resistant mutant (DRM) candidate in future selection experiments that employ naphthyridine-based compounds or other ligands [26] containing chemical extensions that protrude into the solvent-exposed cleft of the intasome. N117Q will be more conformationally restricted and thus is expected to lead to an entropic loss when larger compounds, such as **4f**, are bound to the intasome. Future selection experiments will test this idea. Furthermore, our observation that the residue at position HIV-1 IN_148_ influences ligand binding to a greater extent than other residues was unexpected and revealing. Q148H/K/R are prominent DRMs, arising frequently in viruses derived from patients on both first- and second-generation INSTI therapy [27,28,29,30]. Although the mutation Q148S does not arise in HIV-1 IN (here, it was tested solely for the purpose of comparing with the analogous mutation in PFV IN), the insights gained from our simulations strongly suggest that the nature of the residue at this position will affect ligand binding and selectivity. Specifically, mutations Q148H/K/R should differentially influence the local hydration pattern in the central sub-pocket. Indeed, an analysis of the previously solved PDB structures of the HIV-1 intasomes containing the mutation Q148H (PDB: 8FNP), Q148K (PDB: 8FNN), and Q148R (PDB: 8FNO) indicates that there are distinct hydration patterns within the central sub-pocket. This implies that a careful analysis of the water profile, specifically in the central sub-pocket but also the network as a whole, will be important for future structure-based drug design, as both the configuration and the total number of waters will affect the free energy of ligand binding [31,32]. These insights will extend to the development of third-generation INSTIs [11,26,33].

### 3.2. Atomic Models of HIV Intasomes for Studying INSTI Binding and Drug Resistance

The first INSTIs were introduced into the clinic in 2007 [34]. A mechanistic understanding of their mode of inhibition was elucidated in 2010 with the experimental structures of PFV intasomes containing bound INSTIs [6]. Since then, the PFV intasome has been used extensively for structure-based drug design [4,7,8,35,36], including for the development of second-generation clinically used inhibitors, and rationalizing mechanisms of drug resistance [9]. However, work by two independent laboratories recently revealed that the PFV intasome has limitations and is too divergent from the HIV-1 intasome to properly interpret mechanisms of drug resistance [33] or to be employed in structure-based drug design [11]. On the latter point, we previously observed that the exact same ligand can engage two different intasomes distinctly, but we could not explain the underlying basis of this differential binding mode. Here, we provide such an explanation. Given the increasing emphasis on understanding the mechanisms of HIV-1 resistance to therapy and targeting, specifically, drug resistant viral variants using novel INSTIs [10,26,37], it is thus important to now use experimental structural biology data derived from HIV-1 intasomes for rationalizing mechanisms of resistance and as starting points for structure-based drug design. The growing accumulation of structural biology data defining the conformations of HIV-1 intasomes, including both WT and DRM variants bound to both clinically used and developmental INSTIs, will help to this end.

### 3.3. Utility of the Alchemical R-FEP-R for Predicting Ligand Binding Modes in Challenging Environments

The utility of the alchemical R-FEP-R method for predicting ligand binding modes in challenging environments is demonstrated in this study. We show that this method can accurately compute the relative stabilities of ligand binding modes in the challenging binding site environments found in HIV-1 and PFV intasomes, where ligand-induced side chain and solvent reorganization, as well as configurational entropy, play significant roles.

As observed in Table 1, R-FEP-R calculations are capable of accounting for the relative thermodynamic stabilities of binding modes that differ by approximately 2 kcal/mol, exceeding the accuracy expected from more approximate methods like MM-PB(GB)SA calculations. Compared to physical pathway-based free energy methods such as umbrella sampling or metadynamics, the use of alchemical dual topology in R-FEP-R simplifies the formidable task of choosing reaction coordinates and/or overcoming the large free energy barrier that separates the binding modes. In our work, the two binding modes of **4f** only differ in their dihedral angles. However, it is important to note that different binding modes can involve differences in both internal parameters (such as torsions) and external factors (i.e., ligand translation and orientation). Importantly, the R-FEP-R method is well-suited to address the free energy difference between binding poses that also involve ligand external degrees of freedom. This is achieved by introducing appropriate restraints on ligand translation and orientation in the dual topology setup of the hybrid system. Consequently, we anticipate that the R-FEP-R method will serve as a general solution for rigorously calculating the relative thermodynamic stabilities of multiple ligand binding modes.

## 4. Conclusions

We have investigated the mechanism responsible for the two distinct conformations of a third-generation INSTI **4f** in complex with the PFV and HIV-1 intasomes, associated with different binding modes to these viral enzymes, by applying a novel molecular dynamics-based conformational free energy method, R-FEP-R. The calculated conformational free energy differences between the two binding modes predict that the extended **4f** conformation is favored in the HIV-1 intasome relative to the bent conformer, whereas the bent **4f** conformation is favored in the PFV intasome. Both results agree with experimental structural observations obtained from different sources. Our analysis of the MD simulations of the different binding modes in the two closely related intasomes, supported by additional R-FEP-R free energy calculations which probe the effects of mutations, has identified the crucial role of the central pocket residue Q148 (HIV-1) and S217 (PFV) and, to a lesser extent, the front pocket residues N117 (HIV-1) and Q186 (PFV) in determining the binding modes of **4f** in the two intasomes. This study highlights the importance of ligand-induced protein side chain and solvent reorganization, and the conformational entropy change in ligand binding and helps inform further development of third-generation INISTIs to combat the rapidly emerging HIV-1 variant strains resistant to existing INSTIs.

## 5. Methods and Materials

### System Setup and Simulation Details

The starting structure of the HIV-1−**4f** complex is taken from the Cryo-EM structure of compound **4f** bound to the HIV-1 intasome (PDB 6PUZ) [34] and the starting structure PFV−**4f** is from the crystal structure of **4f** bound to the PFV intasome (5FRO) [4]. For the mutants of the HIV-1 and PFV intasomes studied in this work, the mutation was introduced using the AmberTool pdb4amber, followed by manual adjustment of the side chain dihedral angles. For example, to introduce the N117Q mutant in HIV-1, the dihedral angles of the mutant Q117 were adjusted in accordance with the corresponding residue Q186 in the experimental structure of wild-type PFV.

Molecular dynamics simulations were performed with GROMACS version 2020.3 [38]. The HIV-1 and PFV intasome proteins were modeled with the ff19SB force field [39], the DNA was modeled using the Parmbsc1 force field [40], and the ligand was modeled with the general AMBER force field (GAFF) [41] and AM1-bcc charge models [42]. The accuracy of the force field model can have a significant impact on the calculated free energy [43,44,45]. Here, to properly model the metal ion Mg^2+^ coordination with the protein and ligand, we use a bonded model involving bond, angle, dihedral, electrostatic, and van der Waals terms [46,47] that are parameterized by following the procedure developed by Pengfei Li and Merz groups (Metal Center Parameter Builder (MCPB)). The bonded models [48] treat the interactions between the metal ion and its ligating atoms as covalent bonds. Lin and Wang [49] applied the bonded model through the use of the Seminario method [50] (using the Cartesian Hessian matrix to calculate the force constant) to zinc complexes with the general AMBER force field (GAFF) and demonstrated the effectiveness of the bonded model performed in structural optimizations and molecular dynamics simulations of four selected model systems. The benchmarking simulations by Melse and Zacharias [51] applying the MCPB.py program reproduced the Zn^2+^ sites accurately, including mono- and bimetallic ligand binding sites. To compute force field parameters for the Mg^2+^-associated atoms of IN (Figure S3), we applied the MCPB.py program with GAMESS-US [52] to generate the bond, angle, corresponding force constants, partial charge, and VDW parameters (Table S2).

One intasome unit was placed at the center of a rectangular box and the distance between the solute and the edge of the box was set to be ≥1.0 nm. A 9.4 nm × 12.7 nm × 10.2 nm box containing 30,200 4-point rigid water model (OPC) [53] water molecules, which were added by the Gromacs program, and 4 Na^+^ was used for HIV-1. An 11.7 nm × 11.1 nm × 14.0 nm box containing 48,800 OPC water molecules and 17 Na^+^ was used for PFV. The total number of atoms in the simulation box was ~132,000 for HIV-1 and 206,000 for PFV. Additional Na^+^ and Cl^−^ ions were added to obtain 0.15 M NaCl in the simulation box. An energy minimization was performed to relax the initial system, followed by 100 ps equilibration of the NVT ensemble, 1 ns equilibration of the NPT ensemble, and a 30 ns production run.

The conformational free energy difference between the extended and bent binding modes of **4f** was computed using the R-FEP-R method (Figure 2) [16,54], which uses an alchemical pathway instead of the traditional physical pathway approach to connect the two conformational basins. In this method, the ligand atoms are divided into the shared part and the varying segment (see Figure 1A), with only the latter participating in the conformational change. R-FEP-R uses the dual topology approach in which the hybrid system contains atoms from both end-states with their contributions to the Hamiltonian controlled by a varying λ value. The free energy difference is computed as the sum of the following components, i.e.:

$$\Delta G_{bent\to ext}=\Delta G_{ext\_release}+\Delta G_{bent(V)\_release}+\Delta G_{ext\left(V\right)+bent\to ext+bent\left(V\right)}+\Delta G_{ext(V)\_restrain}+\Delta G_{bent\_restrain}$$

Here, $\Delta G_{bent\_restrain}$ and $\Delta G_{ext(V)\_restrain}$ are the free energies of restraining the real and virtual segments in the initial state (bent), while $\Delta G_{bent(V)\_release}$ and $\Delta G_{ext\_release},$ are the free energies to release the restrains on the virtual and real segments in the final (extended) state. When the force constants of the restraints used are large, the $\Delta G_{ext(V)\_restrain}$ and $\Delta G_{bent(V)\_release}$ are equal in magnitude (and opposite in sign) and cancel out each other [16,55]. $\Delta G_{ext+bent\left(V\right)\to bent+ext\left(V\right)}$ is the free energy of alchemically converting the virtual segment in the initial state into the real one in the final state (under conformational restraints). All three free energy components $\Delta G_{ext+bent\left(V\right)\to bent+ext\left(V\right)}$ , $\Delta G_{ext\_release}$ , and $\Delta G_{bent\_restrain}$ were computed using FEP.

For calculating $\Delta G_{ext+bent\left(V\right)\to bent+ext\left(V\right)}$ , 39 alchemical λ windows were used: λ = 0, 0.000001, 0.000002, 0.000005, 0.00001, 0.00002, 0.00005, 0.0001, 0.0002, 0.0005, 0.001, 0.002, 0.004, 0.01, 0.03, 0.06, 0.1, 0.15, 0.2, 0.29, 0.38, 0.46, 0.54, 0.62, 0.71, 0.8, 0.85, 0.9, 0.94, 0.97, 0.99, 0.996, 0.999, 0.9995, 0.9999, 0.99995, 0.999999, 0.999 9999, 0.99999999, 1.0. At each λ, the equilibration was performed for 1 ns first. The production run was then performed for 10 ns at 300 K. A constant temperature was maintained by a modified Berendsen thermostat [55]. The electrostatic interactions were treated with the particle mesh Ewald (PME) [56] method with a real-space cutoff of 1.0 nm. MD simulations were performed with a time step of 2 fs and energy files were saved every 2 ps.

The number of alchemical λ windows used for computing the release/restraint free energies $\Delta G_{ext\_release}$ and $\Delta G_{bent\_restrain}$ was set to be 23 (λ = 0 0.0002, 0.0005, 0.001, 0.002, 0.003, 0.006, 0.01, 0.02, 0.04, 0.07, 0.1, 0.15, 0.2, 0.25, 0.3, 0.4, 0.5, 0.6, 0.7, 0.8, 0.9, 1.0). Figure S5 shows that the chosen intervals are sufficient for phase–space overlap, with probabilities well above the recommended threshold of 0.03 [57]. For each individual λ state, the equilibration was performed for 1 ns ensemble, followed by a 10 ns production run. The other parameters are the same as above. Convergence was confirmed by analyzing the simulation data in the forward and reverse directions and checking that the calculated free energies agree within the error (Figure S4) [57].

## Figures and Tables

**Table 1:** The conformational free energy difference, $∆G_{bent\to ext\;}^{site}$ , between the bent and extended conformations of **4f** in the two intasomes.

Receptor		$\mathrm{Calculated}\;∆G_{bent\to ext}^{site}$ (kcal/mol)	Predicted Binding Mode
HIV-1 Intasome	Wild Type	−2.9 ± 0.4	Extended
	N117Q	−2.5 ± 0.4	Extended
	Q148S	−0.7 ± 0.3	Mixed
PFV Intasome	Wild Type	2.2 ± 0.4	Bent
	Q186N	1.4 ± 0.4	Bent
	S217Q	−0.4 ± 0.2	Mixed

**Table 2:** Mean values of the side chain torsion angles of Q148 in the **4f**-bound HIV-1 intasome observed in 30 ns MD simulations and those in the cryo-EM structure of the apo HIV-1 intasome.

Structure	χ_1_ N-CA-CB-CG	χ_2_ CA-CB-CG-CD	χ_3_ CB-CG-CD-OE1
4f-bound (extended)	−66.0°	91.5°	42.8°
4f-bound (bent)	−59.7°	145.7°	−120.1°
Apo	−49.3°	103.4°	29.5°

**Table 3:** Average number of water molecules occupying the central cavity observed in 30 ns MD trajectories.

Intasome	Extended 4f	Bent 4f
HIV	5–6	0
PFV	6–7	1–2

## Data Availability

Data are contained within the article and supplementary materials.

## Supplementary Materials

The following supporting information can be downloaded at: www.mdpi.com/xxx/s1, Table S1: RMSD in the positions of C-alpha atoms between the corresponding binding site residues (within 5 Å from any 4f atoms) in the HIV-1 and PFV intasomes. For the two DNA nucleotides that form part of the binding site, the corresponding deviations between the backbone C5’ atoms are shown; Figure S1: Extended (yellow) and bent (green) 4f in the binding site of HIV-1 intasome. To facilitate a conformational transition between the two binding conformations requires both side chain atoms from the protein residues P142 and Y143 to rearrange to avoid steric clash with the sulfonylphenyl moiety of 4f converting from the extended to the bent conformation and vice versa; Figure S2: Nonpolar interactions between the CB of N117 of HIV-1 and Q186 of PFV with the sulfonylphenyl moiety of the extended 4f. The green sphere represents the centroid of the terminal benzene ring of 4f; Figure S3: The HIV-1 IN active site carboxylates Asp64, Asp116, and Glu152 coordinate a pair of Mg2+ ions, which in turn interact with the metal-chelating core naphthyridine ring of the INSTIs 4f (bottom molecule); Table S2: The bond, angle, corresponding force constants, the partial charge, and the VDW parameters generated by MCPB.py program with GAMESS-US for Mg2+ associated atoms in HIV-1 and PFV intasomes; Figure S4: Time evolution of the free energies (with error bars) for the three stages of transformation of 4f from extended to bent bound to PFV Q186N. Data from the last 10 ns of simulations were analyzed both chronologically (“forward”) and in a time-reversed manner (“reverse”); Figure S5. Overlap matrices for the three stages of transformation of 4f from extended to bent bound to PFV Q186N. The element Oij is the probability of observing a sample from state i (ith row) in state j (jth column). The recommended minimum probability for adjacent states (highlighted by thick black lines) is 0.03; Figure S6: 2D ligand-receptor interaction diagram of Extended 4f-HIV-1 (left) and bent 4f-PFV (right) complexes.
