# Supplementary material for: Elucidating the Molecular Determinants of the Binding Modes of a Third-Generation HIV-1 Integrase Strand Transfer Inhibitor: The Importance of Side Chain and Solvent Reorganization
Source: Viruses. 2024 Jan 2;16(1):0. doi: 10.3390/v16010076 (PMC11154245; doi:10.3390/v16010076)
Supplement: Supplementary file 1 [file viruses-16-00076-s001.zip › viruses-2766314-supplementary.pdf]

Supporting Information for "Elucidating the molecular determinants of the binding modes of a third-generation HIV-1 integrase strand transfer inhibitor: The importance of side chain and solvent reorganization"

12/31/2023

*Qinfang Sun[1], Avik Biswas[2,3], Dmitry Lyumkis[2,4], Ronald Levy[1], Nanjie Deng[5]**

(1) Center for Biophysics and Computational Biology and Department of Chemistry, Temple University, Philadelphia, PA 19122

(2) The Salk Institute for Biological Studies, Laboratory of Genetics, La Jolla, CA 92037

(3) Department of Physics, University of California San Diego, La Jolla, CA, 92093

(4) Graduate schools for Biological Sciences, Section of Molecular Biology, University of California, San Diego, La Jolla, CA, 92093

(5) Department of Chemistry and Physical Sciences, Pace University, New York, NY10038

Table S1. RMSD in the positions of C-alpha atoms between the corresponding binding site residues (within 5 Å from any 4f atoms) in the HIV-1 and PFV intasomes. For the two DNA nucleotides that form part of the binding site, the corresponding deviations between the backbone C5’ atoms are shown.

Corresponding residue pairs	RMSD (Å)
N117(HIV)/Q186(PFV)	0.39
Y143(HIV)/Y212(PFV)	0.36
P145(HIV)/P214(PFV)	0.38
Q146(HIV)/Q215(PFV)	0.42
Q148(HIV)/S217(PFV)	0.41
DNA: A36(HIV)/A16(PFV)	0.43
DNA: C35(HIV)/C17(PFV)	1.06

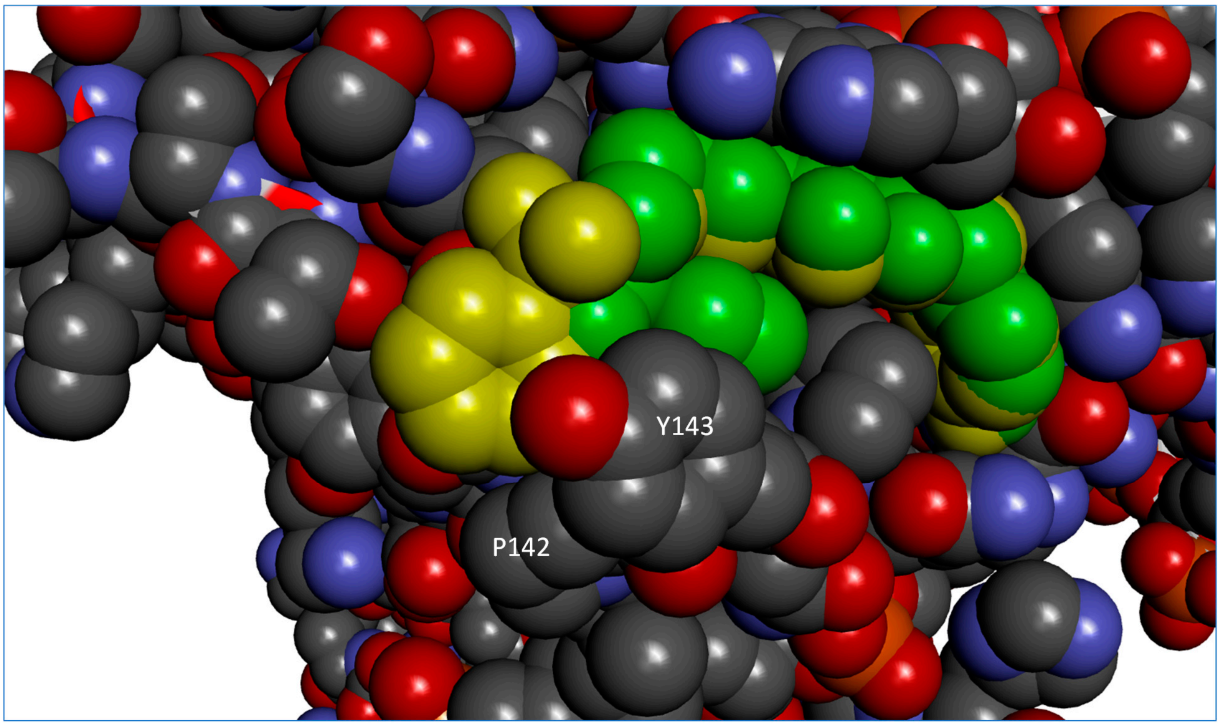

Figure S1. Extended (yellow) and bent (green) 4f in the binding site of HIV-1 intasome. To facilitate a conformational transition between the two binding conformations requires both side chain atoms from the protein residues P142 and Y143 to rearrange to avoid steric clash with the sulfonylphenyl moiety of 4f converting from the extended to the bent conformation and vice versa.

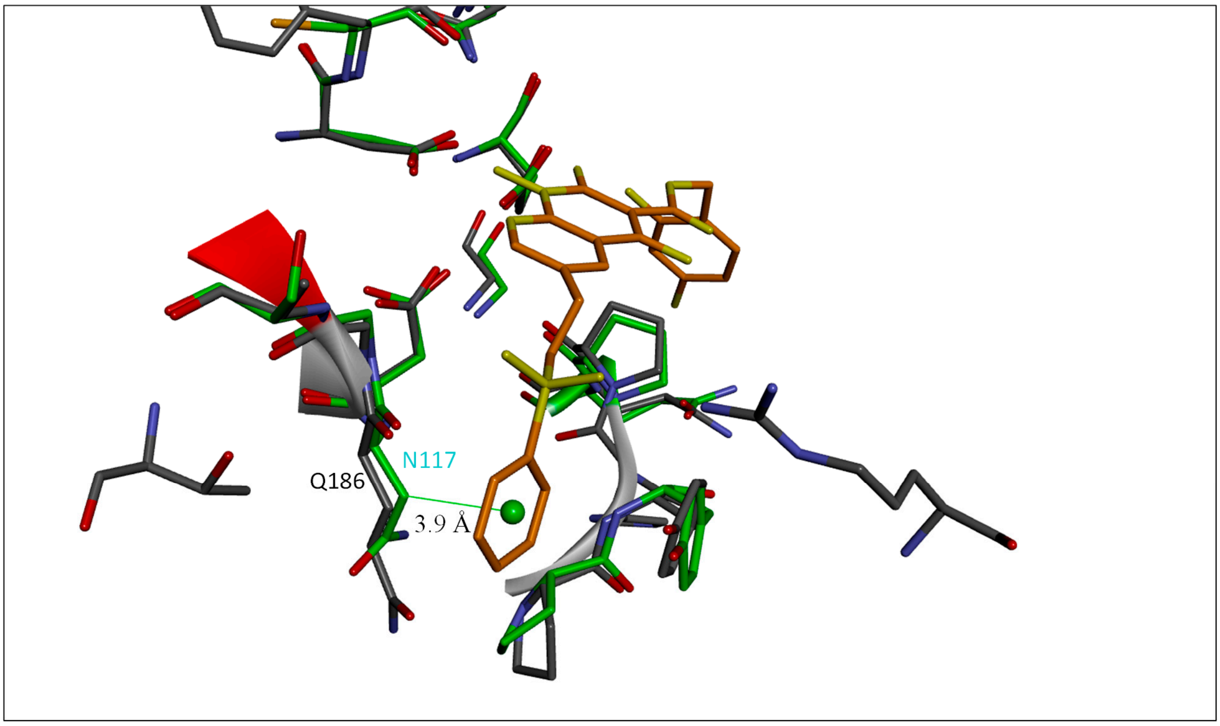

Figure S2. Nonpolar interactions between the CB of N117 of HIV-1 and Q186 of PFV with the sulfonylphenyl moiety of the extended 4f. The green sphere represents the centroid of the terminal benzene ring of 4f.

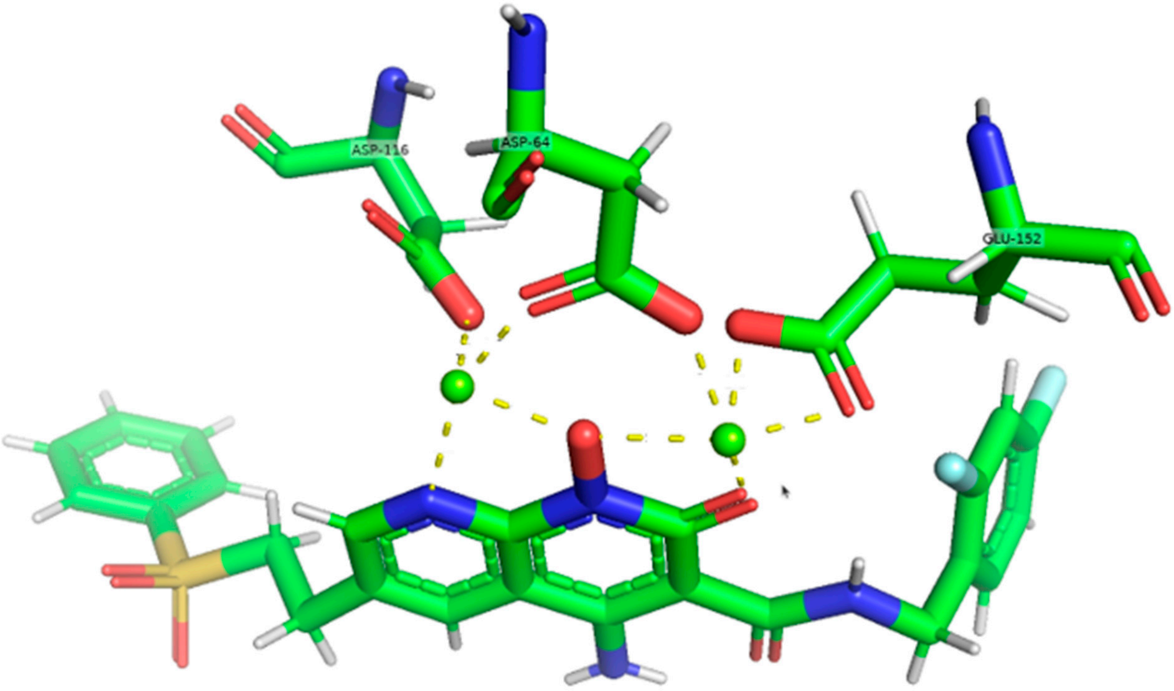

Figure S3. The HIV-1 IN active site carboxylates Asp64, Asp116, and Glu152 coordinate a pair of Mg^{2+} ions, which in turn interact with the metal-chelating core naphthyridine ring of the INSTIs 4f (bottom molecule)

Table S2. The bond, angle, corresponding force constants, the partial charge, and the VDW parameters generated by MCPB.py program with GAMESS-US for Mg^{2+} associated atoms in HIV-1 and PFV intasomes.

Bond parameters for Different Mg^{2+}	Atom1 Name		Atom2 Name	R eq(Å)	Force Const. (kcal/mol*Å^2)
HIV-1 MG1	OD1		MG	1.9747	73.8
	OD2		MG	2.0337	56.7
	MG		OAF	2.04	63.4
	MG		NAV	2.2535	34
HIV-1 MG2	OD2		MG	1.9886	66
	OE2		MG	2.0385	56
	OE1		MG	2.0739	45.5
	MG		OAC	2.0893	47.9
	MG		OAF	2.0794	48.7
PFV MG1	OD1		MG	1.9746	71.5
	OD2		MG	2.0484	51.6
	MG		OAF	2.0398	63
	MG		NAV	2.2665	28.7
PFV MG2	OD2		MG	1.9837	69.4
	OE2		MG	2.044	53.2
	OE1		MG	2.0703	46.5
	MG		OAC	2.0886	46.5
	MG		OAF	2.0785	48.9
Angle parameters for Different Mg^{2+}	Atom1 Name	Atom2 Name	Atom3 Name	Theta eq (Rad)	Force Const. (kcal/mol*Rad^2)
HIV-1 MG1	OD1	MG	OD2	115.51	32.65
	OD1	MG	OAF	91.96	57.16
	OD1	MG	NAV	132.3801	34.49
	CG	OD1	MG	137.3601	51.76
	OD2	MG	OAF	117.5801	56.9
	OD2	MG	NAV	111.37	64.84
	CG	OD2	MG	90.35	112.53
	MG	OAF	NBI	118.9801	73.23
	MG	OAF	MG	125.8301	54.8
	MG	NAV	CAP	120.7801	94.52
	MG	NAV	CBH	120.7801	94.52
	OAF	MG	NAV	73.27	53.5
	OD2	MG	OE1	103.97	69.57
	OD2	MG	OE2	116.8201	31.76

HIV-1 MG2	OD2	MG	OAC	133.0401	36.62
	OD2	MG	OAF	89.03	59.84
	CG	OD2	MG	141.3901	55.29
	OE2	MG	OAC	110.08	52
	OE2	MG	OAF	113.85	71.21
	OE1	MG	OE2	63.26	127.45
	OE1	MG	OAC	94.42	91.26
	OE1	MG	OAF	166.6201	52.12
	CD	OE1	MG	87.84	125.83
	CD	OE2	MG	89.29	119.79
	MG	OAF	MG	125.8301	54.8
	MG	OAC	CBF	118.7601	55.89
	MG	OAF	NBI	114.04	84.05
	OAC	MG	OAF	74	50.48
PFV MG1	OD1	MG	OD2	116.34	32.47
	OD1	MG	OAF	90.14	56.2
	OD1	MG	NAV	143.9901	39.35
	CG	OD1	MG	140.1601	52.54
	OD2	MG	OAF	121.5801	47.21
	OD2	MG	NAV	99.49	51.05
	CG	OD2	MG	89.46	118.25
	MG	OAF	NBI	119.3901	60.94
	MG	OAF	MG	126.8701	49.9
	MG	NAV	CAP	120.6801	98.36
	MG	NAV	CBH	120.6801	98.36
	OAF	MG	NAV	72.93	57.55
PFV MG2	OD2	MG	OE1	104.32	70.41
	OD2	MG	OE2	116.4	17.21
	OD2	MG	OAC	137.5401	17.75
	OD2	MG	OAF	89.38	63.27
	CG	OD2	MG	139.0801	33.99
	OE2	MG	OAC	106.06	43.29
	OE2	MG	OAF	112.39	61.5
	OE1	MG	OE2	63.26	129.05
	OE1	MG	OAC	94.19	84.98
	OE1	MG	OAF	166.1901	62.78
	CD	OE1	MG	88.03	125.33
	CD	OE2	MG	89.06	119.33
	MG	OAF	MG	126.8701	49.9

	MG	OAC	CBF	118.4101	54.8
	MG	OAF	NBI	113.72	76.6
	OAC	MG	OAF	74.06	63.1
VdW parameters and charge for Mg^{2+}	LJ Radius/Å	LJ Depth/kcal/mol	Mass	Charge	
HIV-1 MG1	1.373	0.0118	24.3	1.5414	
HIV-1 MG2	1.373	0.0118	24.3	1.6172	
PFV MG1	1.373	0.0118	24.3	1.508	
PFV MG2	1.373	0.0118	24.3	1.542	

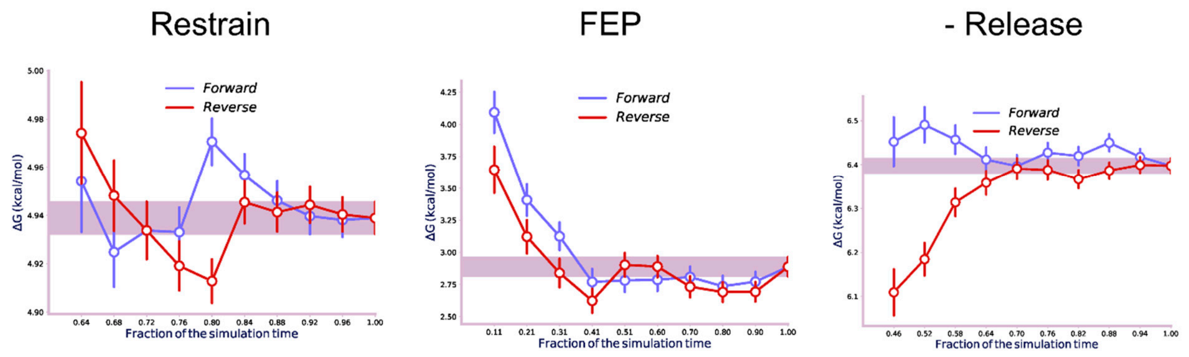

Figure S4. Time evolution of the free energies (with error bars) for the three stages of transformation of 4f from extended to bent bound to PFV Q186N. Data from the last 10 ns of simulations were analyzed both chronologically ("forward") and in a time-reversed manner ("reverse").

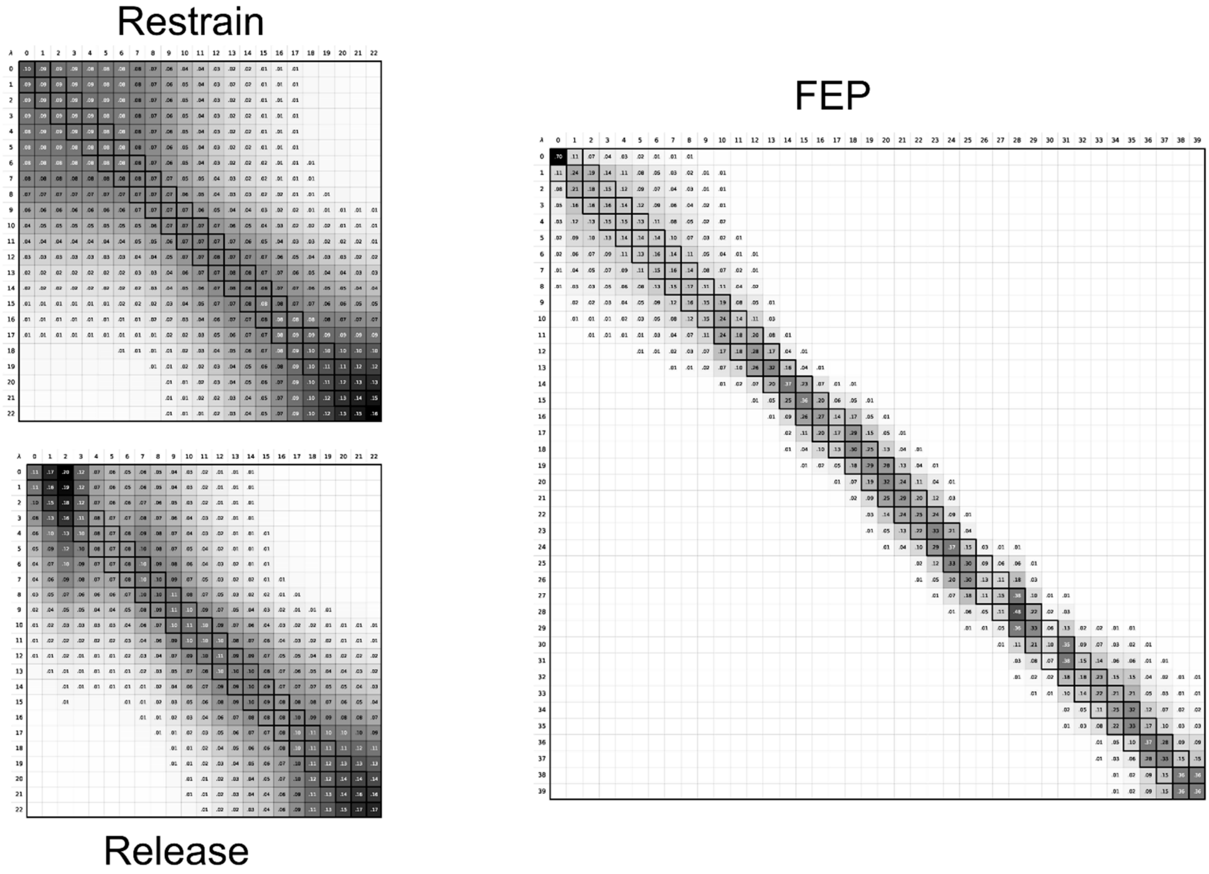

Figure S5. Overlap matrices for the three stages of transformation of 4f from extended to bent bound to PFV Q186N. The element Oij is the probability of observing a sample from state i (ith row) in state j (jth column). The recommended minimum probability for adjacent states (highlighted by thick black lines) is 0.03.

Extended-4f-HIV-1

A

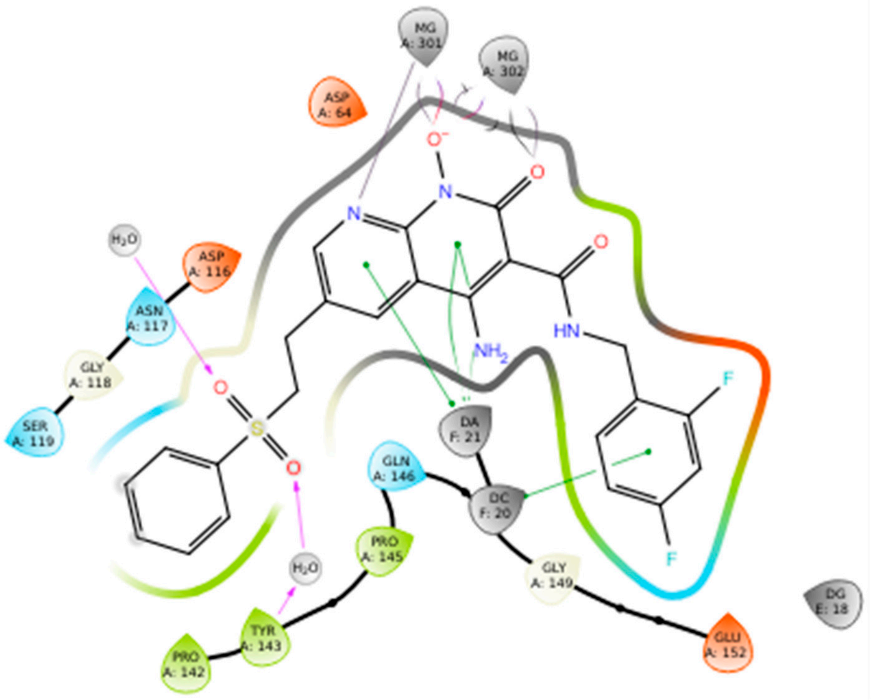

B

Bent-4f-PFV

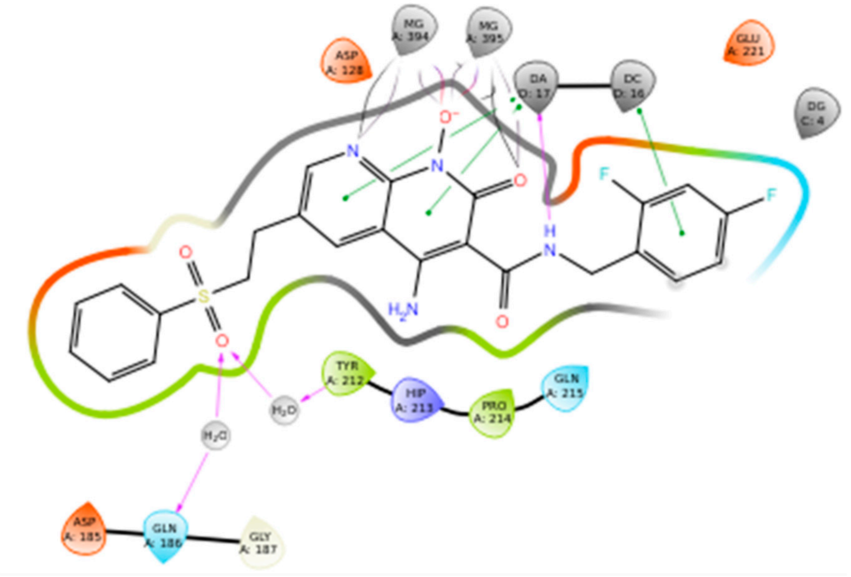

Figure S6. 2D ligand interaction diagrams of (A) Extended 4f-HIV-1 and (B) bent 4f-PFV (right) complexes. The two diagrams are calculated using the Schrodinger Inc's Maestro software.
